# Efficacy of essential oil mouthwash with and without alcohol: a 3-Day plaque accumulation model

**DOI:** 10.1186/1745-6215-12-262

**Published:** 2011-12-15

**Authors:** Enrico Marchetti, Stefano Mummolo, Jonathan Di Mattia, Fabio Casalena, Salvatore Di Martino, Antonella Mattei, Giuseppe Marzo

**Affiliations:** 1 Department of Health Sciences, School of Dentistry, University of L'Aquila, P.le G. Liberatore, Ed. Delta 6, 67100 L'Aquila, Italy; 2 Department of Internal Medicine and Public Health, University of L'Aquila, P.le G. Liberatore, Ed. Delta 6, 67100 L'Aquila, Italy

**Keywords:** Antiplaque agents, chemical plaque control, oral hygiene, essential oils, alcohol, mouthwash

## Abstract

**Background:**

The aim of this study was to evaluate the antiplaque effect of a new alcohol free essential oil mouthwash with respect to a control of an essential oil with alcohol mouthwash, using an *in vivo *plaque regrowth model of 3-days.

**Methods:**

The study was designed as a double-masked, randomized, crossover clinical trial, involving 30 volunteers to compare two different essential oil containing mouthwashes, during a 3-day plaque accumulation model. After receiving a thorough professional prophylaxis at the baseline, over the next 3-days each volunteer refrained from all oral hygiene measures and had two daily rinses with 20 ml of the test mouthwash (alcohol free essential oil) or the control mouthwash (essential oil with alcohol). At the end of the each experimental period, plaque was assessed and the panelists filled out a questionnaire. Each subject underwent a 14 days washout period and there was a second allocation.

**Results:**

The essential oil mouthwash with ethanol shows a better inhibitory effect of plaque regrowth in 3-days than the mouthwash test with only essential oil in the whole mouth (plaque index = 2.18 against 2.46, respectively, p < 0.05); for the lower jaw (plaque index = 2.28 against 2.57, respectively, p < 0.05); for the upper jaw (plaque index = 2.08 against 2.35, respectively, p < 0.05); for the incisors (plaque index = 1.93 against 2.27, respectively, p < 0.05); and the canines (plaque index = 1.99 against 2.47, respectively, p < 0.05).

**Conclusion:**

The essential oil containing mouthwash without alcohol seems to have a less inhibiting effect on the plaque regrowth than the traditional alcoholic solution.

**Trial Registration:**

ClinicalTrials.gov NCT01411618

## Background

The daily removal of supragingival dental plaque is a major factor in the prevention of caries, gingivitis and periodontitis. Proper control of bacterial plaque is obtained through the mechanical removal of the biofilm by the proper use of the toothbrush and floss. However, some studies have shown that the mean time of brushing tooth surfaces is less than that required to obtain a proper cleaning [1] and only 2-10% of the patients use dental floss regularly and effectively [2]. In addition, it has been demonstrated that even after education and motivation of the patient to the proper use of toothbrush and floss, its compliance is reduced with time [3]. The result is the persistence of plaque in some areas, particularly on the interproximal surfaces of teeth. Many studies have demonstrated the effectiveness and usefulness of antiseptic mouthwashes containing active ingredients such as chlorhexidine (CHX) and essential oils (EO) to prevent and control the formation of plaque and gingivitis, when used in addition to mechanical procedures [4-7]. Chlorhexidine is still the gold standard for its antimicrobial action and high substantiveness, but side effects, such as pigmentation, taste alteration and the formation of supragingival calculus limit its continued use [8]. Essential oil (EO) mouthwashes have been used for years as an adjunct to brushing in addressing oral hygiene. Their effectiveness in controlling plaque and gingivitis are well documented in literature [9-14]. They kill microorganisms by destroying their cell walls and inhibiting their enzymatic activity [15,16]. Furthermore, phenolic compounds like EOs are known to interfere with the inflammation process [17,18]. The antibacterial action is particularly effective for the ability of the mouthwash with EOs to penetrate the biofilm [19-21]. The traditional EO mouthwashes contain ethanol, a chemical used to dissolve numerous substances in mouthwashes, including CHX. The concentration of ethanol present in the mouthwash with EOs is more than 20%, sufficient to dissolve the EOs but not enough to carry out a direct antibacterial effect [22,23]. Many aspects against the use of alcohol in mouthwashes, such as its effects on the surfaces of composite restorations [24] and its possible role in the formation of oropharyngeal cancer are being discussed [25,26]. Although a direct correlation of the cause and effect between the occurrence of oropharyngeal cancer and the use of mouthwashes with alcohol [27], has not demonstrated so far, it is considered desirable to eliminate ethanol for use in daily mouthwash, bringing in search of new formulations. Recently, an EOs containing mouthwash without alcohol was introduced on the European market (Daycare, Curaden, Kriens, and Suisse). To our knowledge, to date there are no published data on the effectiveness of this antimicrobial product. The rinsing with this mouthwash can cause fewer side effects but, in contrast, it may be less effective.

The aim of this study was to evaluate the inhibitory properties of a new alcohol free EO containing mouthwash with respect to the traditional mouthwash containing 21.3% ethanol, through a standard 3-days plaque regrowth model.

## Materials and methods

### Study Population

Thirty healthy non-dental student volunteers (13 females and 17 males; age range 18 to 35 years; mean age: 23.9 years) participated in the study. The subjects were recruited through advertisements. All candidates were screened for suitability by the research team. Selection criteria were a dentition with ≥20 evaluable teeth (minimum of five teeth per quadrant), no oral lesions, no severe periodontal problems (no probing depth ≥5 mm), and no removable prostheses or orthodontic bands or appliances. Persons allergic to several mouthwash components were excluded from the study.

All eligible subjects were given oral and written information about the products and the purpose of the study and were asked to sign an informed consent. The study was conducted from June 08, 2010 to September 21, 2010 in the Division of Periodontology, University of L'Aquila, in accordance with the ethical principles originating in the Declaration of Helsinki and consistent with good clinical practices. The study was not commenced until the approval of the ethics committee of ASL 1 Avezzano - Sulmona - L'Aquila was obtained (11/2010/SC).

### Study Design

**Figure 1:**
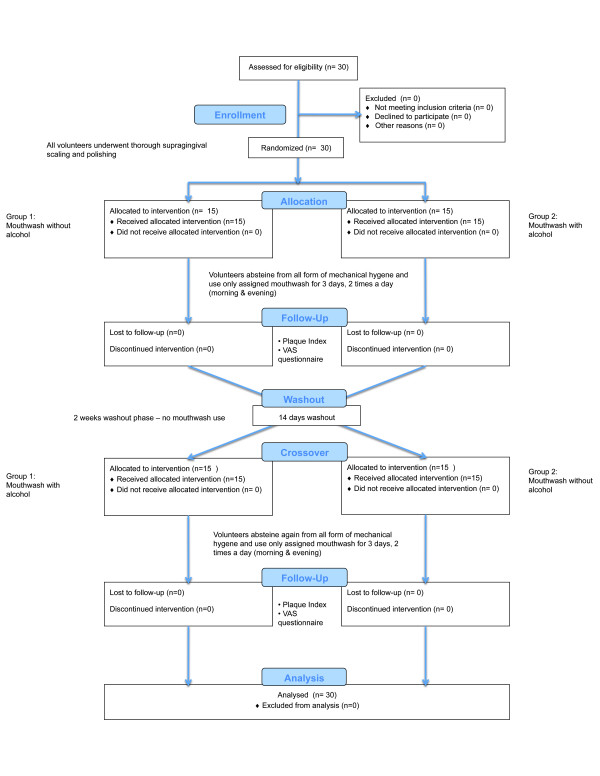
**Flow diagram illustrating the study design**.

The study was designed as a double-masked, two-group crossover randomized clinical trial (Figure 1). On day 1 of each of the 2 study periods, after disclosing the teeth, the volunteers received an oral soft and hard tissue examination and a professional scaling and polishing to remove all calculus, plaque and extrinsic tooth stain. This was performed using hand instruments, mechanical scalers, rotating brushes with polishing paste, and dental floss in the interproximal areas. To ensure that all deposits were removed, a second disclosing episode was carried out.

Volunteers were randomly assigned to the test or control group. Randomization was performed using computer-generated random numbers. A person not directly involved in the research project carried out the allocation of test or control products.

The subjects in the test group received a bottle of mouthrinse containing an alcohol free EO mouthwash^‡^, whereas subjects in the control group received a bottle of mouthrinse containin a traditional alcohol containing EO mouthwash^§^. All bottles with mouthwash were pre-weighed. All participants were instructed to refrain from using any other means of oral hygiene during the experimental period.

All subjects were instructed to rinse twice a day, in the morning and in the evening, with 20 ml solution for 60 seconds. Subsequent rinsing with water was not allowed. Written instructions were provided explaining how to use the mouthwash. Rinsing was performed at home without supervision. To check for compliance, subjects were asked to note the times of day when they rinsed.

After 3 days, all volunteers were disclosed with an erythrosine solution and the plaque in both groups was recorded using the Quigley and Hein index as modified by Turesky et al. [28]. All measurements were carried out under the same conditions by the same blinded investigator (DM). The examiner was trained and calibrated in the plaque scoring system. All returned mouthwash bottles were weighed to calculate the amount of mouth rinse used and to check for compliance. All volunteers received a questionnaire using a visual analog scale (VAS) designed to evaluate their attitudes with regard to the product used. Subjects marked a point on a 10-cm-long uncalibrated line with the negative extreme response (0) at the left end and the positive extreme response (10) at the right end [29].

A washout period of 14 days was instituted between the treatments, when subjects resumed their normal oral hygiene habits; following this 2-week washout period, all subjects underwent again a session of scaling in order to get the plaque index to 0 and the procedures were repeated with subjects using the alternative rinse.

### Statistical Analysis

The population size of the study was calculated *a priori*. Using a power calculator, it was determined that ≥30 subjects were necessary so that a 20% reduction in parameter (hypothesis of plaque reduction tests compared to control) with a 10% standard deviation as clinically relevant, a power (1-β) of ≥ 80% was computed for the two-sided null hypothesis, Ho. The plaque scores were used as the main response variables. Mean plaque values were calculated at the subject level and were considered both in the totality of the oral cavity and for several areas: upper jaw, lower jaw, incisors and canines. The normality distribution of the data was checked with the Shapiro-Wilk test.

Data were analyzed using non-parametric statistics, which are more powerful when the data show a skewed distribution. Thus, the Wilcoxon signed rank-sum test was more appropriate to ascertain the significant differences between the individual rinse solutions to compare the difference between the test and control. Data considering the VAS-scores of the questionnaire were also analyzed using the same test.

All data were registered electronically using Microsoft Excel and statistical analyses were carried out using the statistical package Intercooled Stata I2. All tests were used in the two-tailed version and *p*-values ≤ 0.05 were considered as statistically significant.

## Results

All subjects (N = 30) completed the trial, and there were no missing values. The amounts of mouthwashes used indicated good compliance with the instructions. No adverse events or side effects were reported or observed. The plaque scores for the test and control groups at the end of the experimental period are shown in Table 1. Statistical analysis showed that there was a significant difference in the plaque index between the two groups. In the control group, the mean overall plaque index was 2.46 compared to 2.18 of the test group after the 3-day non-brushing experimental period. The difference of 0.28 between the two groups was statistically significant (p < 0.05). The EO mouthwash with ethanol shows better inhibitory effect of plaque regrowth in 3 days than the mouthwash test of the whole mouth (plaque index = 2.18 against 2.46, respectively, p < 0.05); for the lower jaw (plaque index = 2.28 against 2.57, respectively, p < 0.05); for the upper jaw (plaque index = 2.08 against 2.35, respectively, p < 0.05); for incisors (plaque index = 1.93 against 2.27, respectively, p < 0.05) than for the canines (plaque index = 1.99 against 2.47, respectively, p < 0.05).

**Table 1:** Total Plaque Index (Mean ± SD) for the Test and Control Groups (N = 30)

	EO with alcohol	EO without alcohol	p value*
Overall	2.18 ± 0.39	2.46 ± 0.42	< 0.001

All vestibular	2.51 ± 0.49	2.88 ± 0.58	< 0.001

All lingual	1.86 ± 0.45	1.86 ± 0.57	ns

Incisors	1.93 ± 0.48	2.27 ± 0.59	< 0.001

Canines	1.99 ± 0.46	2.47 ± 0.56	< 0.001

Premolars	2.13 ± 0.41	2.40 ± 0.48	0.003

Molars	2.64 ± 0.49	2.75 ± 0.37	ns

*Using Wilcoxon signed-rank test.

ns: no statistically significant difference between EO with alcohol and EO without alcohol was found.

The subjects completed the questionnaire after each experimental period and the results are shown in Table 2.

**Table 2:** Questionnaire Responses (Mean ± SD) Determined by VAS

Question	Response		EO with alcohol	EO without alcohol	p value*
1) How was the taste of the product?	Very bad	Very good	5.17 ± 2.73	6.6 ± 2.27	ns

2) How long did the taste remain in the mouth after rinsing?	Very long	Very short	5.97 ± 1.71	5.5 ± 2.36	ns

3) How was your taste of food and drinks affected?	Negative change	Positive change	2.3 ± 3.02	2.97 ± 3.27	ns

4) Was the use of the mouth rinse convenient?	Not convenient	Very convenient	5.83 ± 1.68	5.13 ± 2.58	ns

5) What is your opinion about the rinsing time?	Very long	Very short	4.23 ± 2.88	6.5 ± 2.46	0.0025

6) What was your perception of the plaque reduction?	Insufficient	Very efficient	6.07 ± 2.52	4.97 ± 2.72	0.016

*Using Wilcoxon signed-rank test.

ns: no statistically significant difference between EO with alcohol and EO without alcohol was found.

With regard to the subjects' rating of the rinsing time (question 5), the results demonstrated a difference between the test and control groups (6.50 and 4.23 respectively, p < 0.005). This suggest than the rinsing time of EO with alcohol seemed longer than EO without alcohol, probably due to the typical burning effect of EO with alcohol.

They also considered EO with alcohol (VAS 6.07) as more effective in reducing plaque in the mouth compared to the EO without alcohol (VAS 4.97; p < 0.05).

However, with respect to the other questions (taste perception, duration of taste, alteration in taste perception and convenience), the statistically significant differences were not noted between the groups.

## Discussion

Essential oil mouthwashes are used for many years in the prevention and treatment of periodontal disease. The effectiveness of the EO mouthwash in controlling plaque was demonstrated in many clinical trials, both long- and short-term. In short-term studies [30-33], EO significantly reduced plaque accumulation in the 4- and 21-days plaque regrowth models. However, when the EO rinse was compared to the CHX formulation, the latter showed better antiplaque effects. In contrast, recent studies [34,35] have demonstrated that the EO rinse was as effective as the CHX rinse in inhibiting plaque regrowth. The effectiveness of the EO rinse was also demonstrated in long-term studies [5,8,9,11,36] in which it was used as a supplement to mechanical hygiene measures. In those studies, which varied in length from 3 to 9 months, the EO-containing mouthwash significantly reduced plaque accumulation and gingivitis when compared to the placebo. Although, EO formulations were less effective in reducing supragingival plaque accumulation than CHX [9], in contrast a more recent study [12] has demonstrated that the EO mouthwash and the CHX mouthwash have comparable antiplaque and antigingivitis activity. Furthermore, several studies have demonstrated that EO and CHX rinses were equally effective in reducing gingival index scores and the number of bleeding sites [9,12,37]. Finally, in a recent systematic review [38], the authors concluded that although the effects of CHX ensure higher control of plaque, there are no marked differences in the control of gingival inflammation. Therefore, the EO mouthwash appears to be a reliable alternative to the CHX mouthwash in those cases where the dental professional has judged that long-term anti-inflammatory oral care may be beneficial, while for indications where plaque control is the focus, a CHX mouthwash remains the first choice. All the studies reviewed refer to the EO mouthwash with its traditional formulation, containing alcohol. Alcohol is present in many mouthwashes and is used as a solvent of the ingredients and the preservative of the preparation. The antiseptic effect, however, seems to be negligible at the concentrations used in mouthwashes [22]. *In vitro *studies have shown that the alcohol promotes the mucosal penetration of the various carcinogens found in tobacco [39], in addition causing direct damage on the oral mucosa [40]. The bulk of the metabolism of alcohol is carried out in the liver but there is evidence that demonstrates the ability of some oral bacteria to metabolize alcohol to acetaldehyde, a carcinogenic substance [41]. For many years, researchers have been discussing the safety of alcohol in mouthwashes for daily use and several case-control studies suggest the correlation of alcohol use in mouthwashes with oral cancer [27].

Epidemiological studies, however, are often inconsistent and many reviews conclude there are no data demonstrating the direct correlation between alcohol containing mouthwashes and oral cancer [42,43]. Recently, Werner and Seymour [44] have reviewed the two most recent revisions on the role of alcohol in the onset of oral cancer [27,45], stating that there is evidence showing the existence of this association, but these are still weak and inconclusive, and randomized clinical trials would be needed on a large sample to verify this hypothesis. These authors concluded that the benefit of alcohol in mouthwashes is negligible and it may carry a risk of oral cancer, which is difficult to quantify, and so it is preferable not to prescribe or recommend them.

The purpose of this study was to evaluate whether a new alcohol-free mouthwash with EO had the same characteristics as the antiplaque traditional alcohol-containing formulation, used as a control. It used a 3-day non-brushing model that allowed for plaque accumulation that was already used by several authors to evaluate the effects of various mouthwashes [29,46-49]. In addition, it was investigated through a questionnaire that assessed the volunteers' perception in terms of effectiveness, utility, and taste of the products examined.

The results reject the null hypothesis that there are no differences between the mouthwash test without alcohol and the control with alcohol, in favor of the traditional product with alcohol. The difference in efficacy, as assessed by the plaque index, is modest but statistically significant. There were no differences between the two groups in terms of compliance, taste perception, duration of taste, alteration in taste perception and convenience, while the volunteers considered the mouthwash with alcohol more effective in reducing plaque formation.

## Conclusions

This 3-day plaque regrowth study showed that the EO containing mouthwash without alcohol was a less potent plaque inhibitor than the traditional alcohol containing EO mouthwash. It appears that the subjects appreciated the effect on plaque reduction of the traditional mouthwash better.

## Competing interests

The authors declare that they have no competing interests.

## Authors' contributions

EM conceived of the study, participated in its design and in drafting the manuscript, SM participated in the trial design and was involved in drafting the manuscript, JDM carried out all clinical measurement. FC carried out the allocation of test or control products, SDM participated in the draft the manuscript and in coordination of the team, AM performed the statistical analysis, GM has made substantial contribution to the study conception and revised the manuscript. All authors read and approved the final version of the manuscript.

## Footnotes

* Department of Health Sciences, School of Dentistry, University of L'Aquila, Italy.

† Department of Internal Medicine and Public Health, University of L'Aquila, Italy.

‡ Curasept Daycare, Curaden International AG, Kriens, Suisse

§ Listerine, Johnson & Johnson, S. Palomba-Pomezia, Italy.
